# Supporting Shared Decision-making About Surveillance After Breast Cancer With Personalized Recurrence Risk Calculations: Development of a Patient Decision Aid Using the International Patient Decision AIDS Standards Development Process in Combination With a Mixed Methods Design

**DOI:** 10.2196/38088

**Published:** 2022-11-14

**Authors:** Jet Wies Ankersmid, Sabine Siesling, Luc J A Strobbe, Johanna M Meulepas, Yvonne E A van Riet, Noel Engels, Janine C M Prick, Regina The, Asako Takahashi, Mirjam Velting, Cornelia F van Uden-Kraan, Constance H C Drossaert

**Affiliations:** 1 Department of Health Technology and Services Research University of Twente Enschede Netherlands; 2 Santeon Utrecht Netherlands; 3 Department of Research and Development Netherlands Comprehensive Cancer Organisation Utrecht Netherlands; 4 Department of Surgery Canisius Wilhelmina Hospital Nijmegen Netherlands; 5 Department of Quality and Safety Catharina Hospital Eindhoven Netherlands; 6 Department of Surgery Catharina Hospital Eindhoven Netherlands; 7 Department of Internal Medicine Maasstad Hospital Rotterdam Netherlands; 8 Department of Internal Medicine Leiden University Medical Centre Leiden Netherlands; 9 Department of Neurology OLVG Amsterdam Netherlands; 10 ZorgKeuzeLab Delft Netherlands; 11 Dutch Breast Cancer Society Utrecht Netherlands; 12 Department of Psychology, Health & Technology University of Twente Enschede Netherlands

**Keywords:** patient decision aid, PtDA, breast cancer, surveillance, risk information, shared decision-making, SDM

## Abstract

**Background:**

Although the treatment for breast cancer is highly personalized, posttreatment surveillance remains one-size-fits-all: annual imaging and physical examination for at least five years after treatment. The INFLUENCE nomogram is a prognostic model for estimating the 5-year risk for locoregional recurrences and second primary tumors after breast cancer. The use of personalized outcome data (such as risks for recurrences) can enrich the process of shared decision-making (SDM) for personalized surveillance after breast cancer.

**Objective:**

This study aimed to develop a patient decision aid (PtDA), integrating personalized risk calculations on risks for recurrences, to support SDM for personalized surveillance after curative treatment for invasive breast cancer.

**Methods:**

For the development of the PtDA, the International Patient Decision Aids Standards development process was combined with a mixed methods design inspired by the development process of previously developed PtDAs. In the development, 8 steps were distinguished: establishing a multidisciplinary steering group; definition of the end users, scope, and purpose of the PtDA; assessment of the decisional needs of end users; defining requirements for the PtDA; determining the format and implementation strategy for the PtDA; prototyping; alpha testing; and beta testing. The composed steering group convened during regular working-group sessions throughout the development process.

**Results:**

The “Breast Cancer Surveillance Decision Aid” consists of 3 components that support the SDM process: a handout sheet on which personalized risks for recurrences, calculated using the INFLUENCE-nomogram, can be visualized and which contains an explanation about the decision for surveillance and a login code for a web-based deliberation tool; a web-based deliberation tool, including a patient-reported outcome measure on fear of cancer recurrence; and a summary sheet summarizing patient preferences and considerations. The PtDA was assessed as usable and acceptable during alpha testing. Beta testing is currently ongoing.

**Conclusions:**

We developed an acceptable and usable PtDA that integrates personalized risk calculations for the risk for recurrences to support SDM for surveillance after breast cancer. The implementation and effects of the use of the “Breast Cancer Surveillance Decision Aid” are being investigated in a clinical trial.

## Introduction

Follow-up after curative treatment for breast cancer can be subdivided into *aftercare* and *posttreatment surveillance*. *Aftercare* focuses on information provision, guidance, identification, and dealing with complaints, symptoms, and the physical or psychosocial effects of the disease and treatment [1]. The primary aim of *posttreatment surveillance* is the early detection of locoregional recurrences (LRRs) or second primary tumors (SPs) [1]. In the Netherlands, surveillance is currently one-size-fits-all for all patients with curatively treated breast cancer. However, the risks for LRRs and SPs differ per patient [2,3], and surveillance can be personalized to reduce health care and patient burden. Annual physical examination and imaging are recommended for at least 5 years after treatment for a large group of women with a relatively low risk for recurrences. However, for these women, less intensive surveillance is as effective as more intensive surveillance in terms of diagnosis of LRRs and SPs, and overall survival [4,5].

A woman’s personalized 5-year risk for LRRs and SPs after treatment for breast cancer can be estimated using the INFLUENCE nomogram, a validated prediction model [2,3]. Furthermore, patient needs and preferences should be considered when personalizing surveillance. Patients describe trade-offs between burdens, such as the burden of going to the hospital, anxiety, discomfort, and pain because of the examination and benefits such as the reassurance that surveillance can offer [6]. Therefore, the decision regarding the organization of posttreatment surveillance (eg, frequency, duration, and examination) can be seen as a preference-sensitive decision for which shared decision-making (SDM) is identified as the preferred way of decision-making [7].

SDM can be seen as an indicator of quality of care and is being increasingly reported in breast cancer guidelines [8,9]. It can be defined as *“*an approach where clinicians and patients share the best available evidence when faced with the task of making decisions, and where patients are supported to consider options, to achieve informed preferences*”* [10,11]. Within the process of SDM, four steps can be distinguished: (1) the professional informs the patient that a decision is to be made and that the patient’s opinion is important, (2) the professional explains the options and their pros and cons, (3) the professional and the patient discuss the patient’s preferences and the professional supports the patient in deliberation, and (4) the professional and the patient discuss the patient's wish to make or defer the decision, and discuss follow-up [10]. Recent studies show that even though patients are open to SDM for personalized surveillance, it is only rarely applied and information needs remain unaddressed [6,12,13].

Patient decision aids (PtDAs) are evidence-based tools designed to help patients make specific and deliberate choices among various health care options. PtDAs provide evidence-based information and help patients recognize and clarify values that may play a role in decisions [14]. Clear and objective risk information is an essential component of PtDA. General risk information (about groups of patients) is often presented, but this information is difficult to translate to individual cases [15,16]. Nomograms are being increasingly developed to better estimate individual personal risks. However, these nomograms are rarely integrated into PtDAs [17].

This study aimed to develop a PtDA integrating personalized risk calculations regarding the risk of LRRs and SPs to support SDM for personalized surveillance after curative treatment for invasive breast cancer.

## Methods

### Overview

The development of the “Breast Cancer Surveillance Patient Decision Aid” was initiated by Santeon, a group of 7 collaborating top clinical hospitals in the Netherlands. ZorgKeuzeLab was the development and implementation partner. ZorgKeuzeLab has developed and implemented over 25 PtDAs and therefore has high expertise. For the development of the PtDA, the International Patient Decision Aids Standards (IPDAS) development process [18] was combined with a mixed methods design inspired by the development process of PtDAs previously developed in collaboration with ZorgKeuzeLab [19-21]. In the development, eight steps were distinguished: (1) establishing a multidisciplinary steering group, (2) definition of the end users and scope and purpose of the PtDA, (3) assessment of the decisional needs of end users, (4) defining requirements for the PtDA, (5) determining the format and implementation strategy for the PtDA, (6) prototyping, (7) alpha testing, and (8) beta testing.

### Step 1: Steering Group

To start the development process, the initiators established a multidisciplinary steering group consisting of relevant experts, including patients that were curatively treated for invasive breast cancer and health care professionals (HCPs). To ensure broad acceptance and high implementation of the tool to be developed, members of the multidisciplinary steering group represented all stakeholders involved in the decision-making process, had expertise in breast cancer surveillance, and came from different institutions. Patient representation was ensured by inviting the Dutch Breast Cancer Society (BVN) and the Dutch Federation of Cancer Patient Organizations to participate in the steering group. A selection was made of potential steering group members, and approximately 25 potential steering group members were invited by email to participate. The steering group members were to convene during 5 steering group sessions from which the timing and content were determined based on the steps of the development process. The steering group sessions were prepared and led by a small group of steering group members (including authors JWA, RT, and JBM). The aim of each session was evaluated at the end of each session.

### Step 2: End Users, Purpose, and Scope

The end users, purpose, and scope of the PtDA were determined based on consensus discussions among the steering group members, supported by input from decisional needs assessment studies among patients and HCPs. A small group of steering group members set up a proposal for the end users, purpose, and scope, which was presented and discussed in the first steering group session. Related results from decisional needs assessment studies among patients and HCPs were presented to support this discussion.

### Step 3: Decisional Needs Assessment End Users

Two decisional needs assessment studies were set up and performed among 22 patients (patient needs assessment study) and 21 HCPs (HCP needs assessment study) according to the Ottawa Hospital Research Institute’s guidelines [22] to determine the needs regarding SDM about personalized posttreatment surveillance. For both the needs assessment studies, semistructured interviews were conducted between August 2019 and February 2020. The interviews lasted about one hour and were performed by one researcher (JA, PhD Candidate, MSc in Psychology) who was trained in conducting interviews. Female patients who received curative treatment for invasive breast cancer and had completed their primary treatment were eligible to participate in the patient needs assessment study. The interviews with patients focused on the following topics: (1) current information provision about surveillance, (2) current decision-making about surveillance, (3) preferences for decision-making about surveillance, (4) current use and perspectives on the use of information on personal risks for recurrences in decision-making about surveillance, and (5) perspectives on less intensive surveillance in case of low personal risk. HCPs involved in the follow-up after breast cancer were eligible to participate in the HCP needs assessment study. The interviews with HCPs focused on a broad range of preferences regarding decision-making concerning surveillance and the following topics: (1) perspectives on less intensive surveillance for women with low risks for recurrences, (2) attitudes regarding SDM about surveillance, and (3) perspectives on the use of information on personal risks for recurrences in decision-making about surveillance. Transcripts of all interviews were coded by independent coders (JA and CD) and analyzed using the “framework methodology” [23], which consists of a combination of inductive and deductive approaches: in each of the main topics, the coders inductively searched for themes that emerged from the data. Further details regarding the method of the needs assessment studies can be found in 2 previously published papers [6,24].

### Step 4: Requirements

On the basis of the IPDAS minimum standard criteria [25], in combination with steering group discussions and the results of the needs assessment studies, a list of requirements for the PtDA was developed. This list of requirements was used to inform the format and implementation strategy for the PtDA (step 5), prototyping (step 6), and alpha testing (step 7).

### Step 5: Format and Implementation Strategy

The format of the PtDA was determined in consultation with the steering group and was inspired by the 4 steps of SDM and the format of other existing PtDAs [10,19-21,26]. The implementation strategy was determined in the earlier stages of development (before prototyping) to enable optimization of the design and content of the PtDA and to adapt it to the workflow. Furthermore, it would allow for the early identification and addressing of potential implementation issues [27]. The results of an assessment of the follow-up care pathways in the Santeon hospitals [12], successful implementation strategies for existing PtDAs [28], and a web-based self-management app using patient-reported outcome measures (PROMs) to monitor the quality of life which focuses on awareness, willingness, and behavior of both HCPs and patients [29] were used as a basis for the implementation strategy for the PtDA. The final implementation strategy was determined through consultations with the steering group.

### Step 6: Prototyping

On the basis of the results of the needs assessment studies and the determined format, several low-fidelity prototypes were developed during the three cocreative steering group sessions. ZorgKeuzeLab (the development and implementation partner) uses an approach in cocreative design and prototyping, consisting of the following steps: (1) designing the summary sheet, (2) determining the structure and content of the web-based deliberation tool, and (3) designing the handout sheet. The prototypes were discussed, evaluated, and improved (multiple times if needed) by the steering group members to the high-fidelity prototype used for testing. The presentation of personal risks for LRRs and SPs (including uncertainty) in our PtDA was based on the literature on the current best practices for risk presentation in PtDAs [30]. Various risk presentations for the personal risks of LRRs and SPs were considered, discussed, and adapted during consensus discussions in the steering group during the prototyping phase.

### Step 7: Alpha Testing

Alpha testing of the PtDA consisted of (1) checking whether all determined requirements were met, (2) usability and acceptability testing with patients, and (3) usability and acceptability testing with HCPs. The PtDA was checked for all minimal requirements by the authors JA and CD using the list of requirements that were developed by the steering group.

Alpha testing with patients was conducted in May 2020. Eligible participants were female patients who were curatively treated for breast cancer and finished their primary treatment. We strived to include 6-8 patients [31]. The patients were invited to participate through the social media platform of the BVN. A total of 10 patients volunteered for whom 6 participated (all women, aged 44-75 years, mean 54 years). Owing to COVID-19, tests were performed virtually using Microsoft Teams. First, patients were given a handout sheet with a fictitious patient with a specific (fictious) illness, treatment characteristics, and personal risks for recurrences. They were asked to go through the web-based deliberation tool while thinking aloud about their experiences and thoughts. Any observed difficulties or expressed problems were noted by researchers (usability). After this, patients were interviewed about their satisfaction with the content, layout, and perceived usefulness of the PtDA (acceptability). Finally, we asked the patients whether they would recommend the tool to others (acceptability). Patients’ understanding of the risk information was not included in the tests.

Alpha testing of HCPs was performed in May 2020. A total of 14 HCPs, involved in surveillance after breast cancer, participated (6 surgical oncologists, 4 nurse practitioners, 2 medical oncologists, 1 radiation oncologist, and one research nurse). They were selected and approached via email by steering group members. Our aim was to include more HCPs than patients because the PtDA was intended for use in several hospitals and therefore there was a need to explore routes for implementation (how it fits in the workflow) and potential barriers and facilitators for implementation. Alpha testing was conducted through telephone interviews, which were held after the HCPs had gone through the PtDA by themselves. During the interviews, all 3 components of the PtDA were discussed. Furthermore, HCPs were asked about suggestions for improvement regarding the workflow and content of the PtDA (usability), if they would use the PtDA themselves, and whether they would recommend it to others (acceptability).

The alpha testing sessions with patients and HCPs were summarized and analyzed by authors JA, RT, and AT using the framework methodology [20]. The results, including suggestions for improvement, were discussed in the last steering group session, in which decisions were made on the final adaptations.

### Step 8: Beta Testing

Beta testing (field testing) of the “Breast Cancer Surveillance Decision Aid” with patients and HCPs is ongoing in a large clinical trial. The effectiveness and implementation of shared decision-making supported by outcome information among patients with breast cancer (SHOUT-BC) trial is a multiple interrupted time-series design study in which 630 breast cancer patients will be included in 2 conditions (before or after implementation of the PtDA) in 7 top clinical hospitals (Santeon hospitals) over a period of 20 months. Data will be collected at 3 time points using questionnaires: after the consultation in which the decision for the organization of posttreatment surveillance was made and after 6 and 12 months. In addition, 230 consultations between HCPs and patients facing decisions about the organization of surveillance care will be audio recorded and analyzed. Additional data (eg, data on health care use) will be collected from patients’ medical records. The primary outcome will be patient-reported SDM. The secondary outcomes include observed SDM, decisional conflict and regret, fear of recurrence, risk perception, disease perception, and quality of life. More details on the SHOUT-BC trial can be found in the published study protocol [32].

### Ethical Considerations

The studies carried out as part of the development of the PtDA were conducted in accordance with local laws and regulations. The Medical Research Ethics Committees United in Nieuwegein, the Netherlands, confirmed that the studies were not subject to the Medical Research Involving Human Subjects Act (WMO).

## Results

### Step 1: Steering Group

**Figure 1:**
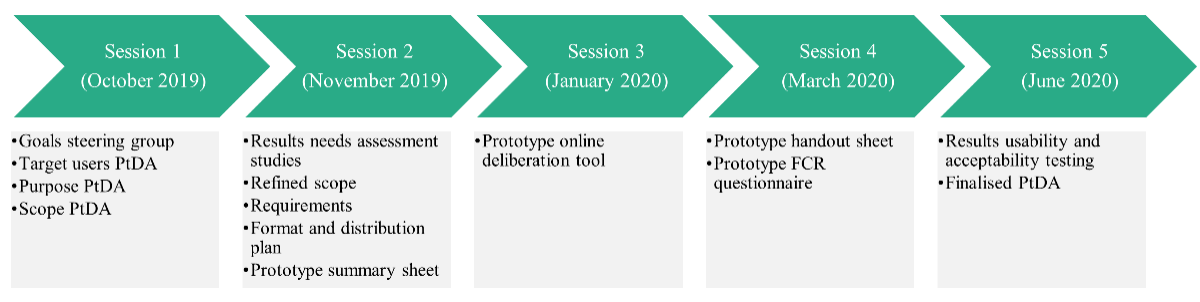
Timing and topics of cocreative steering group sessions. FCR: fear of cancer recurrence; PtDA: patient decision aid.

The established steering group consisted of 15 members: 3 surgical oncologists, 1 medical oncologist, 1 radiation oncologist, 2 nurse specialists, 2 patient advocates, 1 patient representative, 1 operational manager of an oncology department, 1 clinical epidemiologist, 2 health psychologists, and 1 communication scientist with experience in SDM. The development process was facilitated by 2 project leaders, the general director, and a user experience expert from ZorgKeuzeLab. An overview of all the steering group members is provided in Multimedia Appendix 1. The steering group convened during regular cocreative steering group sessions between October 2019 and June 2020. In Figure 1, the timing and topics of each steering group session are displayed. The predetermined aims were achieved for each session.

### Step 2: End Users, Purpose, and Scope

The end users of the developed PtDA were women curatively treated for invasive breast cancer after finalizing their primary treatment. Women who were treated palliatively or those with a genetic disposition related to breast cancer were excluded as end users because of differing follow-up care pathways. Male patients, women diagnosed with noninvasive breast cancer, and women who received neoadjuvant systemic treatment were excluded because the risk prediction model (INFLUENCE nomogram) that is integrated within the PtDA is not suitable for calculating their risks for recurrences.

A discussion point regarding the purpose and scope of the PtDA was whether it should entail personalization of surveillance and aftercare (because of the intertwinement of both in clinical practice) or surveillance alone. The steering group eventually agreed that the PtDA should be specifically aimed at decision-making about surveillance and not aftercare after breast cancer because of the more dynamic nature of aftercare. Although regular surveillance moments can be planned according to the steering group, aftercare should be organized in a more flexible manner. For example, through monitoring of patients’ needs through PROMs and by personalizing care on the outcomes of these PROMs. Therefore, the main purpose of the PtDA is to support patients and their HCPs in SDM for personalized surveillance. The decisions that are supported within the scope of the PtDA are the decisions about the frequency of surveillance, the duration of surveillance, the examination or examinations performed during surveillance, and the way of contact with the HCP (eg, face-to-face or teleconsultation).

### Step 3: Decisional Needs Assessment End Users

The decisional needs assessment among patients revealed that SDM regarding posttreatment surveillance is not often practiced. Patients expressed a wish for more SDM and were open to the use of personalized information on risks for recurrences in this process. However, patients indicated that they sometimes experienced an “internal conflict” between rationale (eg, a low risk for recurrences) and feelings or emotions (fear of cancer recurrence [FCR]), resulting in a high need for reassurance. The HCP needs assessment study revealed that most HCPs supported SDM regarding surveillance and were also positive about using personalized information on risks for recurrences. HCPs indicated some common misconceptions among patients that should be addressed in the PtDA (eg, that patients think that surveillance is primarily aimed at the detection of distant metastasis and the overestimation of the value of physical examination during surveillance consultations). Specific information needs, preferences, and prerequisites for SDM about personalized posttreatment surveillance were gathered and translated into requirements for the PtDA (see *Step 4: Requirements*). More detailed results of the needs assessment studies can be found in 2 previously published papers [6,24].

### Step 4: Requirements

The list of requirements for the PtDA developed by the steering group based on the IPDAS minimal criteria, steering group discussions, and the results of the needs assessment studies are displayed in Textbox 1.

**Requirements for the breast cancer surveillance patient decision aid (PtDA). Only the requirements that emerged from the needs assessment studies or steering group discussions are mentioned. More general International Patient Decision Aids Standards criteria were considered the baseline requirements.**

**Information on surveillance and options:**

1. The PtDA informs on the difference between aftercare and surveillance after breast cancer.
2. The PtDA informs on the aim of surveillance including that surveillance is not aimed at active surveillance for distant metastasis.
3. The PtDA informs on the options for the organization of surveillance for each decision modality (frequency, duration, examinations, way of contact with the health care professional [HCP]) including the advantages and disadvantages.
4. The PtDA informs on the limited added value of physical examination in the detection of locoregional recurrence (LRRs) and second primary tumors (SPs).
5. The PtDA informs on the potential added value of self-examination in the detection of LRRs and SPs and on how to perform self-examination.
6. The PtDA informs patients that they can receive aftercare when the frequency of surveillance is less intensive.
7. The PtDA informs on who to contact in case of complaints or worries.

**Probabilities:**

1. The PtDA informs on personal risks for LRRs and SPs.
2. Personal risks for LRRs and SPs should be displayed both verbally (in words) and visually (in a diagram, including visual information about levels of uncertainty of the prediction).
3. The PtDA informs on the factors on which personal risks for LRRs and SPs depend.

**Methods for clarifying and expressing patients’ considerations and preferences:**

1. The PtDA gives insight in the patients’ own level of fear of cancer recurrence and facilitates conversations about experienced fear of cancer recurrence with HCPs.
2. Patients should be able to read about and reflect on other women’s choices and experiences regarding surveillance.
3. The PtDA facilitates clarification of patient preferences and considerations for the organization of surveillance (value-clarification exercise).

**Guidance in deliberation and communication:**

1. HCPs should be able to indicate the available options for the maximum duration of surveillance and the options for examinations.
2. The PtDA facilitates for patients to test their knowledge on the most important aspects of surveillance.
3. The PtDA facilitates for patients to indicate their role-preference for the shared decision-making process regarding personalized surveillance.
4. The PtDA facilitates patients to list any remaining questions that they might have for their HCP.

### Step 5: Format and Implementation Strategy

**Figure 2:**
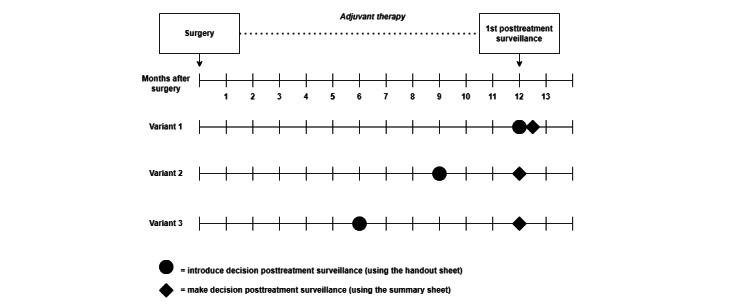
Overview of 3 variants for integration of “Breast Cancer Surveillance Decision Aid” in care pathways.

The steering group determined that the PtDA would consist of 3 components supporting all 4 steps in the SDM process [10]. Each component is described in detail in *Step 6: Prototyping*.” The results of an assessment of the organization of surveillance in the Santeon hospitals were used to determine how the PtDA would fit in the follow-up care pathways [12]. Three different variants for the integration of the PtDA into the care pathways were identified. An overview of the 3 variants is shown in Figure 2. The HCP who introduces the decision about surveillance and who makes the final shared decision about surveillance with patients differs per hospital.

An implementation strategy was developed to implement the PtDA in clinical practice. The implementation strategy consists of the following components:

- Creating support for using the PtDA by cocreation, including both HCPs and patients, and by customizing the PtDA for each hospital (eg, by applying the hospital logo);
- Documenting the current pathways in each hospital to find the best way to incorporate the PtDA [12];
- Informing and involving all HCPs in the care pathway by means of an information meeting, and by offering the possibility to follow the e-learning courses “SDM with patients” and “Applying outcome information in SDM”;
- Giving HCPs the opportunity to practice conversational skills with actors in group training on “SDM and the use of outcome information”;
- Providing an instructional meeting on the use of the PtDA in clinical practice (eg, on how to introduce and discuss it), including reports on experiences of other HCPs and patients who have used the PtDA before;
- Follow-up on the implementation by practical support in clinical practice, a reporting tool to keep track of the implementation rate of the PtDA, and a refresher module of the received conversational skills training program;
- Close monitoring of progress and stimulating implementation of the PtDA by a local ambassador.

### Step 6: Prototyping—the Three Components of the PtDA

**Figure 3:**
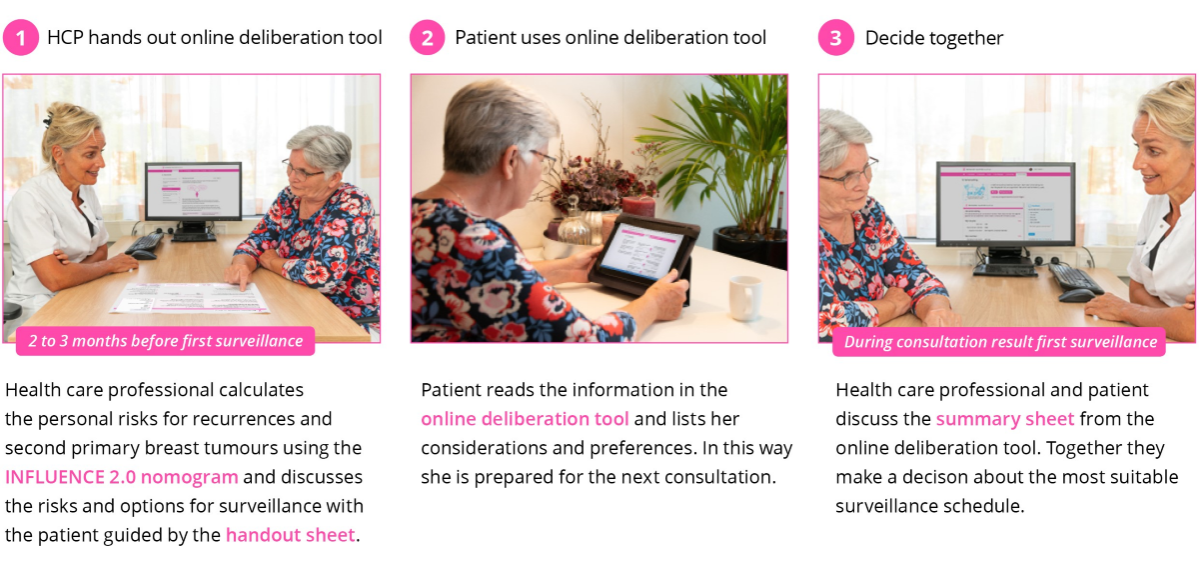
Three components of the Breast Cancer Surveillance Decision Aid. HCP: health care professional.

Several low-fidelity prototypes were developed within the cocreative working group sessions. The high-fidelity prototype that was developed and used for alpha and beta testing consisted of 3 main components (Figure 3) that are described as follows: (1) a handout sheet, (2) a web-based deliberation tool, and (3) a summary sheet.

#### Component 1: The Handout Sheet

**Figure 4:**
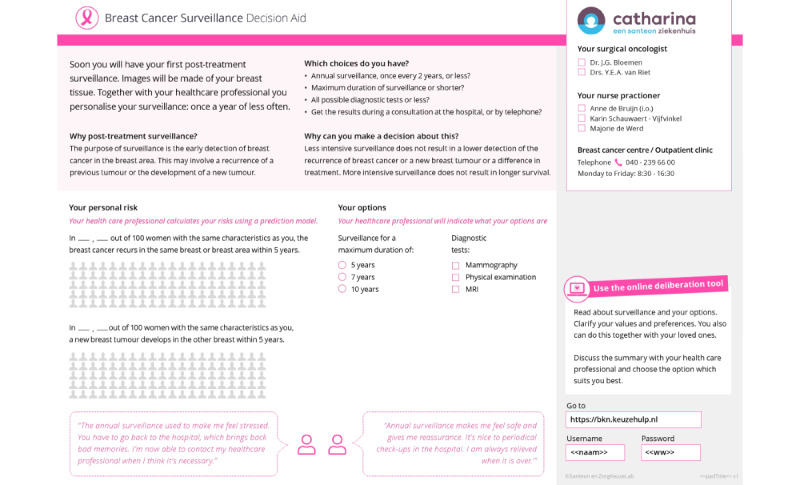
Handout sheet.

Component 1 consists of a handout sheet with which the HCP explains why the patient can co-decide about surveillance and what the options are (eg, frequency, imaging, duration, and preferred contact with HCPs). The handout sheet supports step one and step 2 of the SDM process. The HCP enters the required patient, tumor, and treatment characteristics in the web-based INFLUENCE nomogram [2], which the 5-year risks for LRRs and SPs after treatment for breast cancer can be estimated. This risk is visualized on the handout sheet. The handout sheet also contained the login code and password for the web-based information and deliberation tool. In the PtDA, we make use of a personal login code for several reasons: (1) patients can decide with whom they share the information that they enter the PtDA, (2) patients can access the PtDA and the information that they entered at all times on any device without having to start over, and (3) the login code can be linked to a specific institution that enables implementation measurements (eg, the number of logins per institution). Figure 4 shows the handout sheet.

#### Component 2: The Web-Based Deliberation Tool

Component 2 consists of a web-based information and deliberation tool for women and their caregivers to go through at home at their own pace and time. The web-based deliberation tool supports the second and third steps of the SDM process. The content of the web-based deliberation tool was written at the B1 level of the Common European Framework of Reference for Languages; therefore, it is comprehensible for most patients. The web-based deliberation tool consists of seven modules: (1) your situation, (2) information about surveillance, (3) a quiz, (4) your considerations, (5) your preferences, (6) a questionnaire, and (7) a summary.

**Figure 5:**
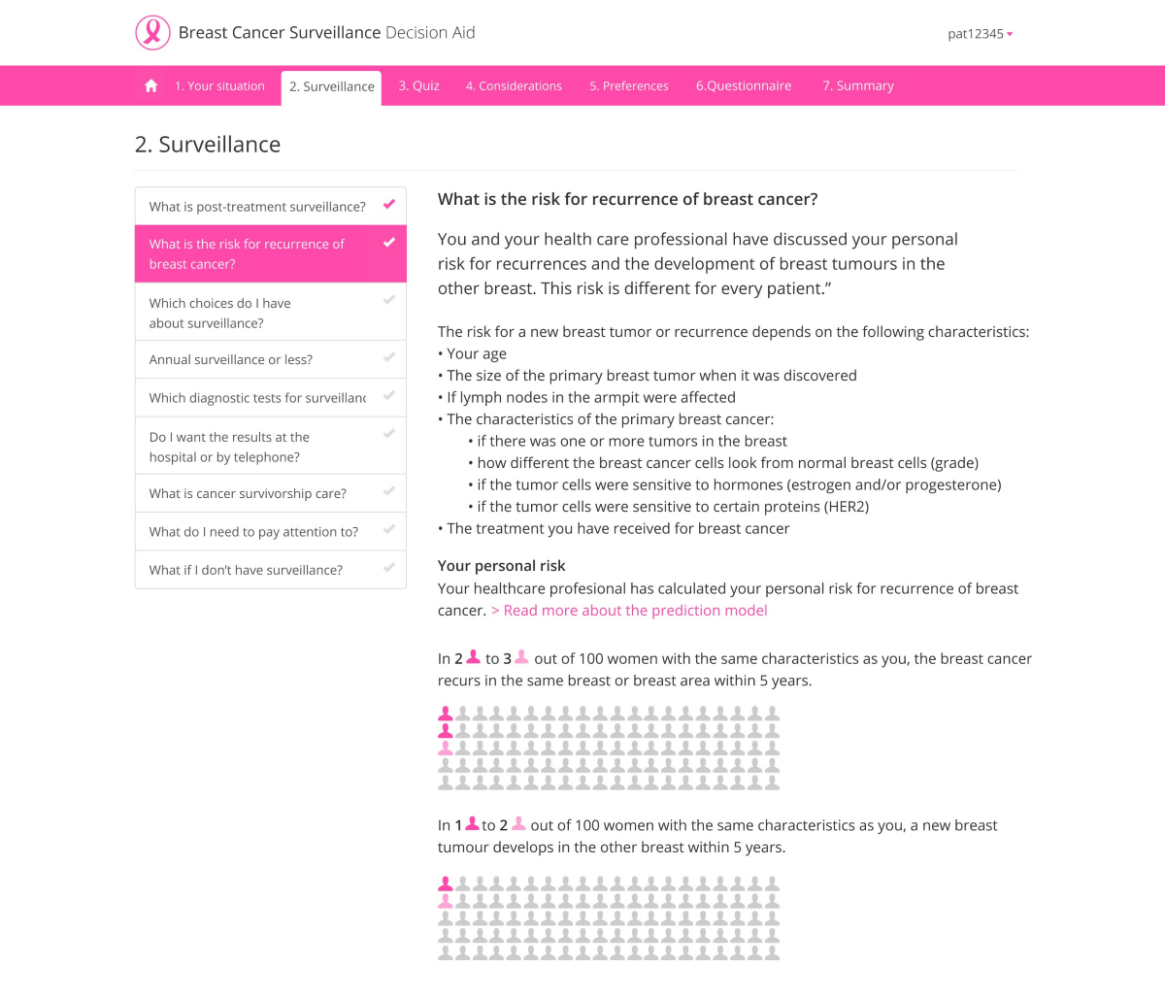
Web-based deliberation tool—module 2 information on risks for locoregional recurrences and second primary tumors.

**Figure 6:**
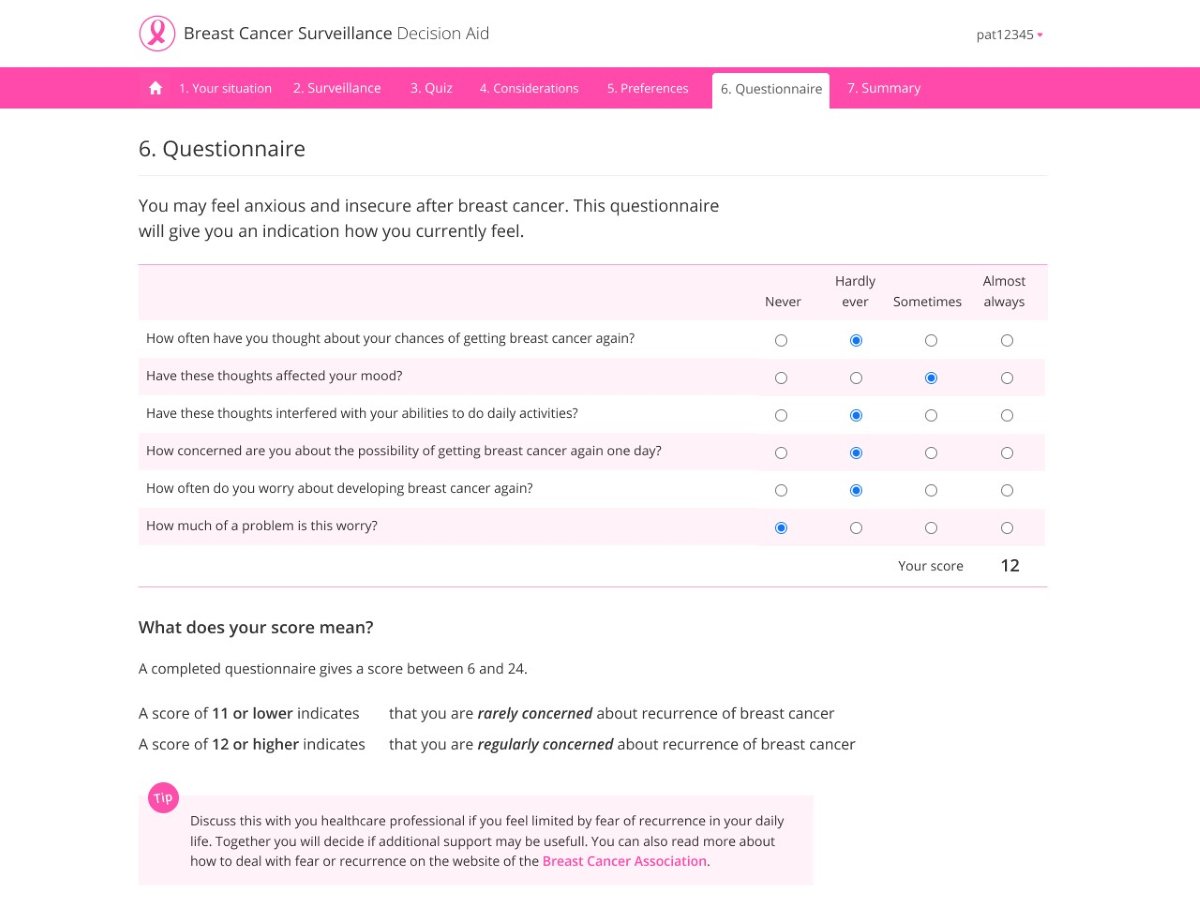
Web-based deliberation tool—module 6—fear of cancer recurrence questionnaire.

In module 1, patients copy their risks for LRRs and SPs and options for the maximum duration and imaging modalities from the handout sheet to the web-based deliberation tool. Module 2 consists of several pages with information about surveillance (structured based on a set of frequently asked questions), the risks for LRRs and SPs, and different options for surveillance and aftercare after breast cancer. Module 3 consists of a knowledge quiz with 3 questions about misconceptions about surveillance with real-time feedback on the answers given. In module 4, patients are presented with a value-clarification assignment with 6 trade-offs on various aspects of surveillance. In module 5, patients can indicate their preference for the options applicable to them for surveillance. In module 6, women are asked to complete the 6-item Cancer Worry Scale questionnaire. This validated questionnaire is meant to assess and screen for FCR in patients that were curatively treated for invasive breast cancer [33]. The input is processed in real time and linked to tailored feedback on individual outcomes (based on validated cut-off scores), including comprehensive self-care advice (tips and tools). This questionnaire has been added to the web-based deliberation tool because the needs assessment studies and usability tests showed that patients regularly experience an “internal conflict” between rationale (low risk) and feelings or emotions (FCR). Because of this conflict, some patients indicated that they would still opt for more intensive surveillance than required for “reassurance.” Many women with breast cancer experience FCR. By integrating the questionnaire, we aim that patients and HCPs can discuss any FCR and that HCPs can provide reassurance, tips for dealing with FCR, or refer the patient to another HCP (eg, a psychologist). In module 7, a summary is generated using the data that the patient has entered (patient preferences, considerations, and FCR score). Figure 5 displays a screenshot of the information on the risk for LRRs and SPs in module 2 of the web-based deliberation tool. Figure 6 shows the questionnaire on FCR in module 6 of the web-based deliberation tool.

#### Component 3: The Summary Sheet

**Figure 7:**
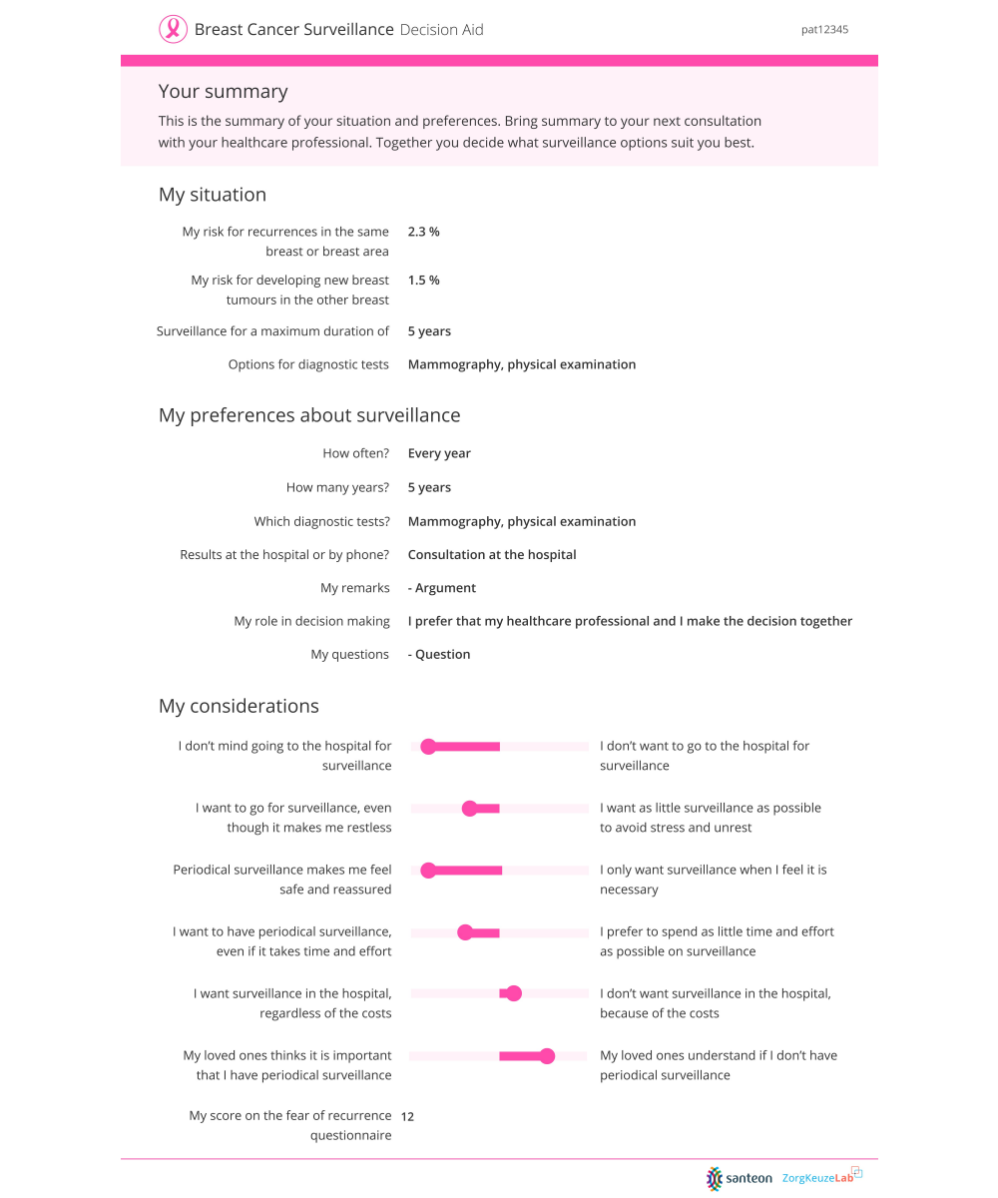
Summary sheet.

Component 3 consists of a summary sheet containing women’s preferences, considerations, and PROM results on fear of recurrence. The sheet can be used by the patient and HCP in the consultation to support step 3 and step 4 of the SDM process and contains all the information that the patients have given as input in the web-based deliberation tool. Figure 7 shows the summary sheet for a fictitious patient.

### Step 7: Alpha Testing

The “Breast Cancer Surveillance Decision Aid” was checked on the requirements established by the steering group (Textbox 1). All requirements were fulfilled.

The patients who were involved in the usability test were positive about the usefulness of the PtDA and would recommend it to other patients (acceptability). The patients indicated that they felt well informed and that they experienced the opportunity to clarify their considerations and preferences regarding surveillance as positive. The patients encountered very few usability issues and found that PtDA was easy to use. However, they found that some of the texts in the PtDA were too extensive.

The HCPs are also positive regarding the PtDA. Most of them indicated that the time was right for personalization of surveillance and that they saw the added value of the PtDA in informing patients and making them more conscious about their options for surveillance (acceptability). HCPs wanted to emphasize the value of self-examination and discuss the limited efficacy of physical examinations by HCPs in PtDA. They also felt that there should be more space to make notes on the discussion with the patient on the handout sheet.

On the basis of the collected feedback, several adaptations were made: (1) more space was created on the handout sheet to make notes, (2) texts within the web-based deliberation tool were shortened where possible, and (3) the descriptions of self-examination and physical examinations by HCPs in the web-based deliberation tool were altered.

### Step 8: Beta Testing

The beta testing (field testing) of the “Breast Cancer Surveillance Decision Aid” is currently ongoing within the SHOUT-BC trial [32].

## Discussion

### Principal Findings

In cocreation, using a step-wise mixed method approach, we developed a PtDA integrating information on patients’ personalized risks for LRRs and SPs to support SDM for personalized surveillance after curative treatment for invasive breast cancer. Development took place according to the IPDAS development process in combination with a mixed methods research design based on the development process of previously developed PtDAs [19-21]. Relevant experts, including patients and HCPs, were involved in development through steering group participation, participation in needs assessment studies, cocreative prototyping, and alpha and beta testing. Our studies revealed a list of requirements that were transferred to the prototype. Alpha testing revealed that all requirements (including the IPDAS minimum standard criteria) for the PtDA were met, and patients and HCPs found the PtDA acceptable and usable. Beta testing is currently ongoing. Throughout the development, we learned some lessons that will be discussed below.

### Comparison With Previous Work

Our PtDA is one of the first to integrate outcome data. We integrated 2 types of outcome data: (1) individual PROMs data on FCR to support structural exploration and consideration of FCR levels and (2) personal risk information based on aggregated clinical data on LRRs and SPs. The results of our study showed that it is feasible to integrate outcome data into the 3-component structure of the PtDA, as both patients and HCPs were positive about the final prototype. Outcome data are expected to accelerate the implementation of SDM by strengthening the motivation of HCPs to apply SDM and empowering patients to engage in SDM [34]. During the steering group discussions, we debated whether a certain value of personal risk or FCR should prescribe a specified pathway in PtDA. However, for this time, we decided that the decision was only to be used as a source of information and not as a guideline because the evidence regarding the most adequate surveillance for specific risk groups needs to be extended. However, it remains interesting to examine whether such pathways are effective in PtDAs.

A challenge in supporting SDM using outcome data is to present data that are readily available to patients in a meaningful manner [16]. The presentation of personal risks for LRRs and SPs (including uncertainty) in our PtDA was based on the literature on the current best practices for risk presentation in PtDAs [30]. Various risk presentations for the personal risks for LRRs and SPs were considered, discussed, and adapted during consensus discussions in the steering group, in which both patients and HCP participated. Although the presentation of personal risks did not cause problems in the alpha testing phase, we did not measure the patients’ understanding of the outcome data. In the beta testing phase, which is currently ongoing, we, therefore, decided to make audio recordings of consultations in which the PtDA is used, to examine how patients interpret and react to hearing their personal risks for recurrences, the FCR PROM score, and the questions they ask. For future research regarding the integration of outcome data into SDM support tools, we recommend testing patients’ understanding of the outcome data during the alpha testing phase.

Within the development of the “Breast Cancer Surveillance Decision Aid,” we have seen the importance of early development of an implementation strategy within the development process. Where (shared) medical decisions were made in one consultation, it is almost inherent to (the steps of) the SDM process, and thus the implementation of a PtDA to split the decision-making process into 2 consultations. This is especially true in the case of a complex decision that requires significant information processing or involves complex information such as outcome data. For successful implementation of PtDAs, it is important that the PtDA fits into the existing system or clinical pathway [35]. For our PtDA, the results of an assessment of the organization of surveillance in the Santeon hospitals were used to determine how the PtDA would fit in the follow-up care pathways [12]. Three different variants for the integration of the PtDA in the care pathways were identified. HCPs and decision support developers should realize that the implementation of PtDAs almost always requires a change in the flow of the care pathway. By assessing the care pathway in each hospital [12] and by determining the implementation strategy in the early stages of development, we could optimize the design and flow of the PtDA to the workflow in the hospitals. Furthermore, this allowed us to identify and anticipate potential implementation issues. In the original IPDAS development process model, attention to the implementation of PtDAs is limited [18,35]. However, recent research has shown that attention for and a successful implementation of are essential for the effectiveness of the developed PtDA [26,27]. We recommend considering implementation as a central part of the development of PtDAs.

During the development of the PtDA, we learned that dividing the steering group into multidisciplinary groups to perform rapid prototyping (during the steering group sessions), followed by a discussion of the prototypes with the complete steering group, enables all steering group members to actively participate in the design of the PtDA. Especially in the design of the outcome data in the PtDA (personal risks for LRRs and SPs and the PROM regarding FCR), this was beneficial for the development process as patients and HCPs were part of the steering group, and they could discuss how the outcome data would benefit them the most within the SDM process. Cocreation is not explicitly mentioned in the IPDAS criteria [18,25], but we recommend that it should be part of the development of every decision-support tool.

ZorgKeuzeLab uses an approach in the cocreative design and prototyping of PtDAs, consisting of the following steps: (1) designing the summary sheet, (2) determining the structure and content of the web-based deliberation tool, and (3) designing the handout sheet. This means that we started designing the last component of the PtDA. This made it easier to stay focused on the scope and relevant content requirements of the PtDA (see also *Step 6: Prototyping* under *Methods*). Therefore, we recommend using this approach for the development of future PtDAs in a similar format.

### Limitations

However, the developmental process of the PtDA has some limitations. First, because we recruited patients for usability and acceptability testing through the social media platform of the BVN, we encountered relatively young patients who may have had more experience with computers and potentially the use of risk information. Second, because of COVID-19, usability and acceptability testing was performed digitally, during which we may not have been able to observe all relevant usability and acceptability aspects, such as the use of the handout sheet and the summary sheet in clinical practice. However, patients and HCPs were satisfied with the web-based deliberation tool and the linkages with the handout and summary sheets in general. Finally, we did not measure patients’ understanding of the outcome data provided during alpha testing. However, in the beta testing phase, audio recordings of consultations in which the PtDA is used are analyzed, and specific attention is given to how patients interpret and react to the provided outcome data and to the questions that they have.

### Conclusions

In conclusion, we developed an acceptable and usable PtDA to support SDM for personalized posttreatment surveillance after breast cancer. The implementation and effects of the use of the “Breast Cancer Surveillance Decision Aid” are being investigated in a clinical trial [32].

## Appendix

Multimedia Appendix 1 Steering group members.

## Abbreviations

BVN: Dutch Breast Cancer Society
FCR: fear of cancer recurrence
HCP: health care professional
IPDAS: international patient decision aids standards
LRR: locoregional recurrence
PROM: patient-reported outcome measure
PtDA: patient decision aid
SDM: shared decision-making
SHOUT-BC: shared decision-making supported by outcome information among patients with breast cancer
SP: second primary tumor
