# Supplementary material for: Supporting Shared Decision-making About Surveillance After Breast Cancer With Personalized Recurrence Risk Calculations: Development of a Patient Decision Aid Using the International Patient Decision AIDS Standards Development Process in Combination With a Mixed Methods Design
Source: JMIR Cancer. 2022 Nov 14;8(4):e38088. doi: 10.2196/38088 (PMC9706380; doi:10.2196/38088)
Supplement: Multimedia Appendix 1 [file cancer_v8i4e38088_app1.docx]

**Multimedia Appendix**

**Multimedia Appendix 1: Overview steering group members**

The multidisciplinary steering group for the development of the PtDA consisted of the following members:

- Y.E.A. van Riet, surgical oncologist at Catharina Hospital
- L.J.A. Strobbe, surgical oncologist at Canisius Wilhelmina Hospital
- T. van Dalen, surgical oncologist at Diakonessenhuis
- E.J.M. Siemerink, medical oncologist at ZGT
- M. van Hezewijk, radiation oncologist at Radiotherapie Groep
- C. Bandel, nurse practitioner at Medisch Spectrum Twente
- D. Jans-van den Heuvel, nurse practitioner at Canisius Wilhelmina Hospital
- A. Kemp, patient advocate at Dutch Breast Cancer Patient Association
- C. van Bavel, patient advocate at Dutch Breast Cancer Patient Association
- M. Velting, patient representative at Dutch Breast Cancer Patient Association
- J.M. Jenje, operational manager oncology department at OLVG
- S. Siesling, clinical epidemiologist, professor ‘outcomes research and personalised cancer care’ at the University of Twente and senior researcher at the Netherlands Comprehensive Cancer Centre
- C.H.C. Drossaert, health psychologist and associate professor at University of Twente
- C.F. van Uden-Kraan, communication scientist and program manager at Santeon
- J.W. Ankersmid, health psychologist and PhD candidate at Santeon and University of Twente

Facilitators of the development process:

- R. The, general director at ZorgKeuzeLab
- A. Takahashi, user experience designer at ZorgKeuzeLab
- J.M. Bode-Meulepas / C. Kerkhofs, projectleader department Quality & Safety at Catharina Hospital
